# Supplementary material for: Microglial ferroptotic stress causes non-cell autonomous neuronal death
Source: Mol Neurodegener. 2024 Feb 5;19:14. doi: 10.1186/s13024-023-00691-8 (PMC10840184; doi:10.1186/s13024-023-00691-8)
Supplement: Supplementary file 1 — Additional file 1: Supplementary Table 1. Case information of ALS-affected spinal cord samples. Supplementary Figure 1. Immunoblots for proteins examined in human and mouse spinal cord. Supplementary Figure 2. Iron content of human, ALS-affected spinal cord. Supplementary Figure 3. Gene expression changes in human, ALS-affected spinal cord. Supplementary Figure 4. GPX4 in spinal cord of human ALS cases and Gpx4(-/-) mice. Supplementary Figure 5. Microglial ferroptosis. Supplementary Figure 6. Retention of neurotoxic factor(s) by 30 kDa MWCO filter. Supplementary Figure 7. Gene expression changes used to monitor neurotoxic glial activation in response to ferroptotic stress in glial cells. Supplementary Figure 8. Isolating astrocytes from mixed glial cultures and gene expression changes in response to treatment with RSL3 or RSL3 plus iron. Supplementary Figure 9. Glutathione and gene expression changes in spinal cord of SOD1G37R mice compared to non-transgenic littermates. Supplementary Figure 10. Changes in cellular expression of LPCAT3 in SOD1G37R and Gpx4(-/-) mice. Supplementary Figure 11. Protective activity of CuII(atsm) in vitro. Supplementary Figure 12. Gene expression changes in glial cultures treated with inducers of ferroptosis and the metallocomplex CuII(atsm). Supplementary Figure 13. Effect of CuII(atsm) on glutathione and gene expression changes in SOD1G37R mice. Supplementary Figure 14. Analysis of PCA for gene expression changes in human ALS-affected spinal cord compared to SOD1G37R mice and RSL3-treated glial cultures. [file 13024_2023_691_MOESM1_ESM.docx]

**Microglial ferroptotic stress causes non-cell autonomous neuronal death**

Supplementary Material

**Supplementary Table 1.** Case information of ALS-affected spinal cord samples.

**Supplementary Figure 1.** Immunoblots for proteins examined in human and mouse spinal cord.

**Supplementary Figure 2.** Iron content of human, ALS-affected spinal cord.

**Supplementary Figure 3.** Gene expression changes in human, ALS-affected spinal cord.

**Supplementary Figure 4.** GPX4 in spinal cord of human ALS cases and *Gpx4*(-/-) mice.

**Supplementary Figure 5.** Microglial ferroptosis.

**Supplementary Figure 6.** Retention of neurotoxic factor(s) by 30 kDa MWCO filter.

**Supplementary Figure 7.** Gene expression changes used to monitor neurotoxic glial activation in response to ferroptotic stress in glial cells.

**Supplementary Figure 8.** Isolating astrocytes from mixed glial cultures and gene expression changes in response to treatment with RSL3 or RSL3 plus iron.

**Supplementary Figure 9**. Glutathione and gene expression changes in spinal cord of SOD1^G37R^ mice compared to non-transgenic littermates.

**Supplementary Figure 10.** Changes in cellular expression of LPCAT3 in SOD1^G37R^ and *Gpx4*(-/-) mice.

**Supplementary Figure 11.** Protective activity of Cu^II^(atsm) *in vitro*.

**Supplementary Figure 12.** Gene expression changes in glial cultures treated with inducers of ferroptosis and the metallocomplex Cu^II^(atsm).

**Supplementary Figure 13.**  Effect of Cu^II^(atsm) on glutathione and gene expression changes in SOD1^G37R^ mice.

**Supplementary Figure 14.** Analysis of PCA for gene expression changes in human ALS-affected spinal cord compared to SOD1^G37R^ mice and RSL3-treated glial cultures.

**Supplementary Table 1. Clinical details and analyses for study ALS and control cases.**

**
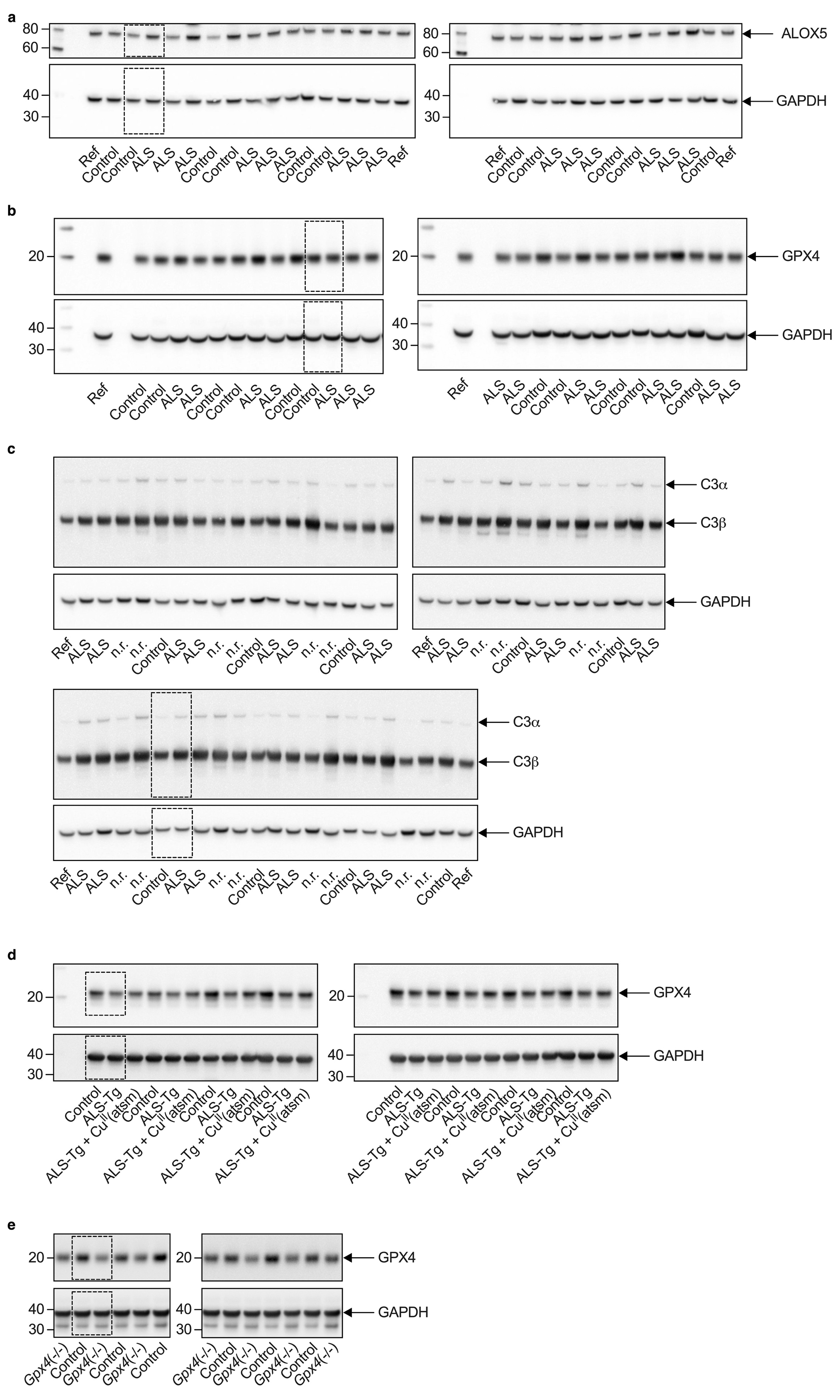
**

(See figure on previous page.)

**Supplementary Figure 1. Immunoblots for proteins examined in human and mouse spinal cord.**

**(a-c)** Immunoblots for ALOX5, GPX4 or C3 protein (as indicated) and GAPDH loading control from human ALS-affected spinal cord. **(d)** Immunoblots for GPX4 protein and GAPDH loading control in spinal cord of SOD1^G37R^ mice (ALS-Tg) treated with or without Cu^II^(atsm) or non-transgenic littermates (Control). **(e)** Immunoblots for GPX4 protein and GAPDH loading control in spinal cord of *Gpx4*(-/-) or control mice. Each lane is an individual case or animal. Dashed boxed regions indicate representative sections displayed in **Fig. 1h** (**a**, ALOX5), **Supplementary Fig. 4b** (**b**, GPX4), **Fig. 4a** (**c**, C3), **Fig. 5f** (**d**, GPX4) and **Supplementary Fig. 4e** (**e**, GPX4). Ref, reference sample; n.r., not relevant to this study.

**
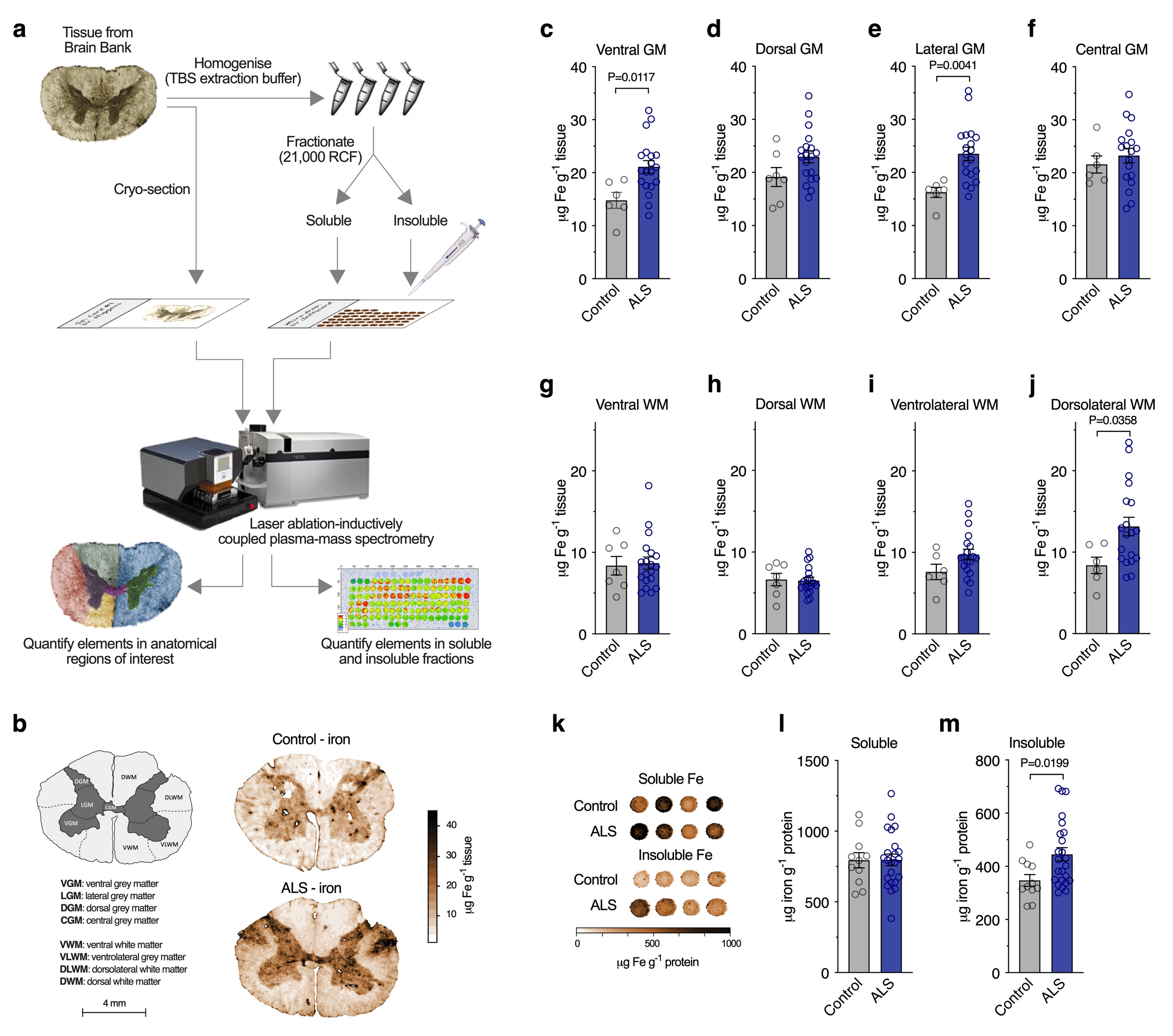
**

**Supplementary Figure 2. Iron content of human, ALS-affected spinal cord.**

**(a)** Procedure for direct elemental quantitation of iron in human spinal cord samples. Cryo-sectioning followed directly by laser ablation inductively coupled plasma-mass spectrometry (LA-ICP-MS) enables *in situ* quantitation. Prior homogenisation in TBS-based extraction buffer and centrifugation enables quantitation of soluble and insoluble partitioning. **(b)** Anatomical map illustrating regions of interest for *in situ* quantitation of iron and representative heatmaps for iron in spinal cord sections (reproduced from **Fig. 1a**). **(c-j)** Iron concentration in indicated anatomical regions of interest determined by LA-ICP-MS. **(k)** Representative images of iron in “micro-droplets” of TBS-soluble and -insoluble fractions of human spinal cord homogenates. **(l,m)** Concentration of iron in TBS-soluble and -insoluble fractions of human spinal cord homogenates. Data points (**c-j**,**l**,**m**) represent individual control or ALS cases. Error margins are S.E.M. and P values indicate significant differences.

**
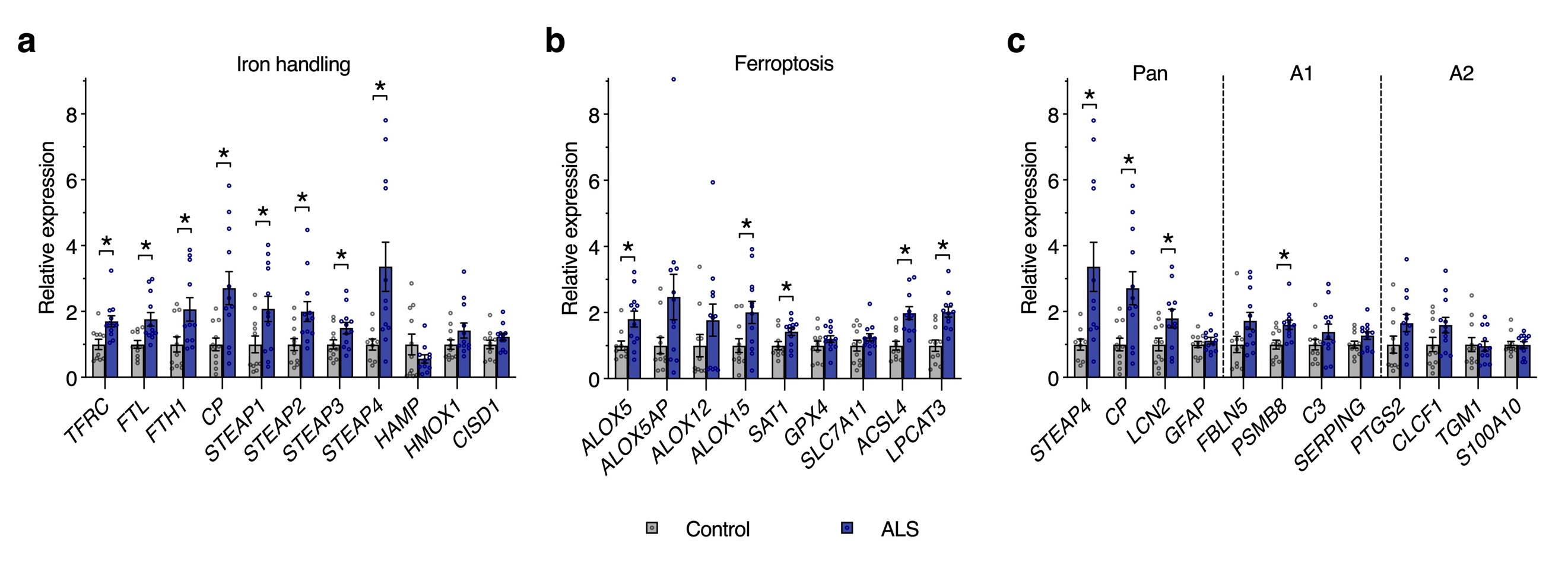
**

**Supplementary Figure 3. Gene expression changes in human, ALS-affected spinal cord.**

Relative expression changes (determined by qPCR) for genes associated with **(a)** iron-handling mechanisms, **(b)** ferroptosis, and **(c)** neurotoxic glial activation, with the latter highlighting selected markers designated for pan, A1 and A2 activation. These data are presented in heatmaps shown in **Fig. 1d, 1i** and **4b** respectively. Asterisks illustrate significant differences (P<0.05) between ALS and respective control. Data points represent individual control or ALS cases. Error margins are S.E.M.

**
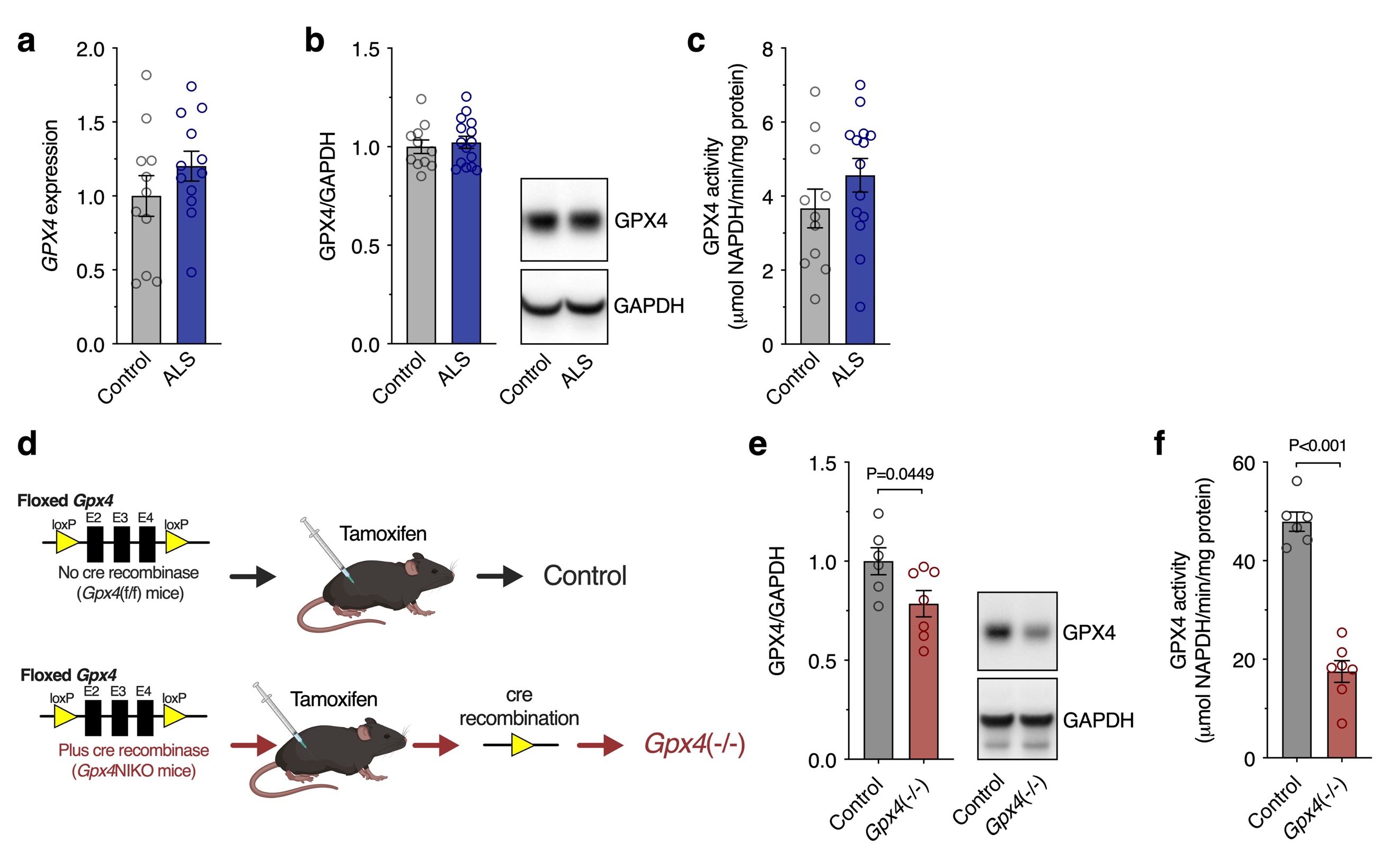
**

**Supplementary Figure 4. GPX4 in spinal cord of human ALS cases and *Gpx4*(-/-) mice.**

**(a)** *GPX4* gene expression in human spinal cord tissue determined by qPCR. **(b)** GPX4 protein levels in human spinal cord tissue determined by western blot. **(c)** GPX4 enzyme activity in human spinal cord tissue determined by rate of RSL3-sensitive, phosphatidylcholine hydroperoxide dependent NADPH consumption. **(d)** To validate the GPX4 activity assay, *Gpx4*(-/-) mice were generated as shown. **(e)** GPX4 protein levels in spinal tissue from control and *Gpx4*(-/-) mice determined by western blot. **(f)** GPX4 enzyme activity in control and *Gpx4*(-/-) mouse spinal cord determined by rate of RSL3-sensitive, phosphatidylcholine hydroperoxide dependent NADPH consumption. Data points represent individual control or ALS cases (**a-c**), or individual animals (**e**,**f**). Error margins are S.E.M. and P values indicated significant differences.

**
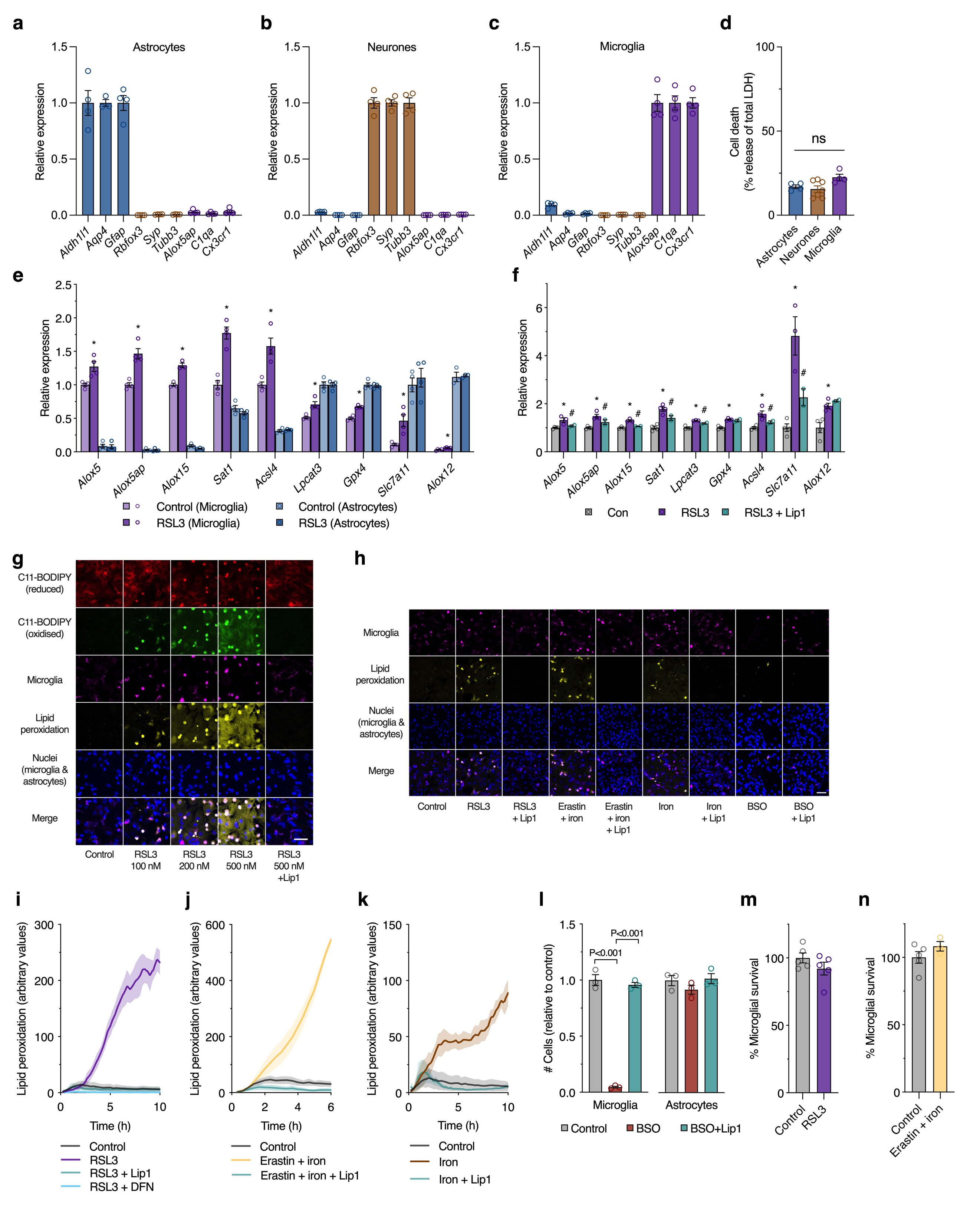
**

**Supplementary Figure 5. Microglial ferroptosis.**

Relative expression of cell type marker genes (determined by qPCR) by primary murine cultures of **(a)** astrocytes, **(b)** neurones and **(c)** microglia. **(d)** Cell death (LDH release) of astrocyte, neurone and microglia cultures in absence of treatments. **(e)** Transcripts associated with ferroptosis in isolated primary murine cultures of microglia or astrocytes after treating with or without RSL3 (25 nM; 8 h), normalised to highest control expression. **(f)** Changes in transcripts associated with ferroptosis in cultured microglia and protective effect of Lip1. These data are presented in heatmap shown in **Fig. 2h**. **(g)** Lipid peroxidation (oxidized:reduced, yellow) assessed using the ratiometric lipid peroxidation probe C11-BODIPY in mixed glial cultures treated with indicated concentrations of RSL3 showing astrocytic lipid peroxidation only at higher RSL3 concentrations and mitigation with liproxstatin-1 (Lip1; visualised in **Supplementary Video 1**). **(h)** Lipid peroxidation in mixed glial cultures treated with alternate inducers of ferroptosis and the protective effect of Lip1 (visualised in **Supplementary Videos 2 & 3**; RSL3 100 nM). **(i-k)** Time dependent changes in microglial lipid peroxidation assessed in mixed glial cultures treated with RSL3, erastin and iron, or iron alone, and mitigation with ferroptosis inhibitors Lip1 or deferiprone (DFN; visualised in **Supplementary Video 2**; lines represent mean of n=3-4 for cells treated with ferroptosis inducers and controls; n=1-4 for cells also treated with Lip1 or DFN). **(l)** Number of surviving microglia and astrocytes in mixed glial cultures treated with BSO and the protective activity of Lip1 (visualised in **Supplementary Video 3**). **(m-n)** Microglial survival in mixed glial cultures treated with RSL3 (100 nM) or erastin and iron determined by transcript analysis of microglial-specific genes. Microglia (magenta) detected with Dylight 649-labelled isolectin in **g**, **h**. Iron in **h**, **j**, **k**, **n** is ferric ammonium citrate. Data points represent independent cultures (**a-f**,**l-m**). Error margins are S.E.M. Significant differences (P<0.05) between control and RSL3 are indicated by asterisks and between RSL3 treated with or without Lip1 by hash symbols (**f**), or by P values in **l**. Scale bar (**g**,**h**) = 50 μm. ns, not significant.

**
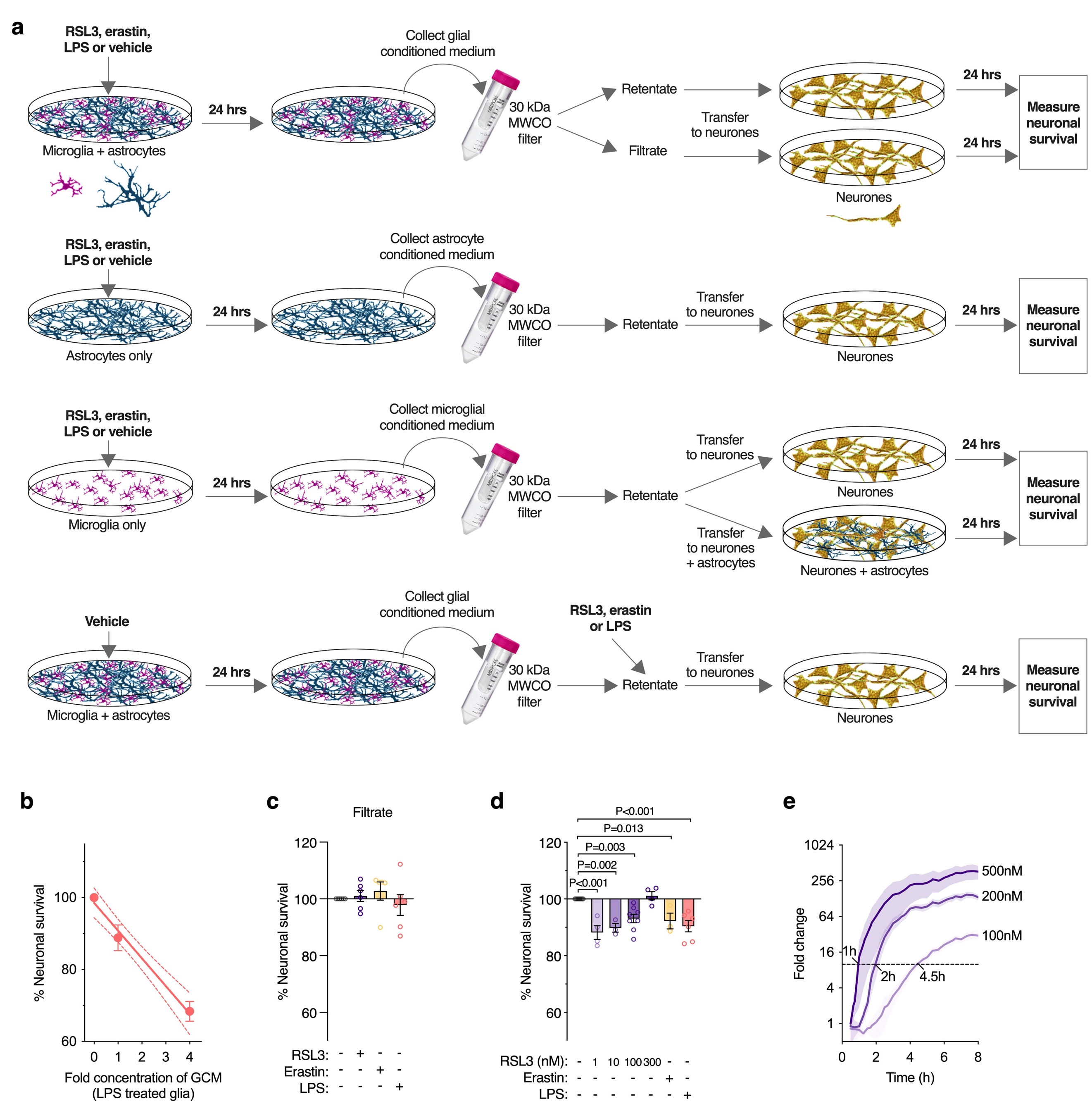
**

**Supplementary Figure 6. Retention of neurotoxic factor(s) by 30 kDa MWCO filter.**

**(a)** Procedure for exposing neurones to glial conditioned medium. RSL3, erastin, LPS or vehicle control are added to either mixed glial cells (microglia and astrocytes), astrocytes or microglial cultures for 24 hrs, as depicted. Conditioned medium from treated glial cultures is passed through a 30 kDa molecular weight cut off (MWCO) filter, then the concentrated retentate or filtrate is transferred to neurones or neurone-astrocyte cultures. As a control, RSL3, erastin or LPS are added to vehicle-treated glial conditioned medium after it is removed from the mixed glial culture. **(b)** Survival of neurones (MTT reduction) relative to concentration of glial conditioned medium. 0 represents glial conditioned medium without LPS treatment; 1 represents neat glial conditioned medium collected from LPS-treated glia; 4 represents 30 kDa MWCO filter retentate diluted into treatment medium to a final concentration equivalent to 4-fold concentration of the neat glial conditioned medium from LPS-treated glia. Data points are mean of n=8-11 independent cultures. Dashed lines represent 95% confidence intervals for linear regression. **(c)** Survival of neurones (MTT reduction) after treating with 30 kDa MWCO filtrate of conditioned medium from mixed glial cultures treated with RSL3 (100 nM), erastin or LPS.  **(d)** Conditioned media from isolated microglia treated as in **a** is toxic to neurone-astrocyte cultures. **(e)** Extent and rate of lipid peroxidation in mixed glial cultures is dose-dependent. Data derived from **Supplementary Video 1**. Lines represent mean of n=2-3 independent cultures. Dashed line represents appearance of conspicuous lipid peroxidation. Data points in **c**,**d** represent independent cultures. Error margins represent S.E.M.

**
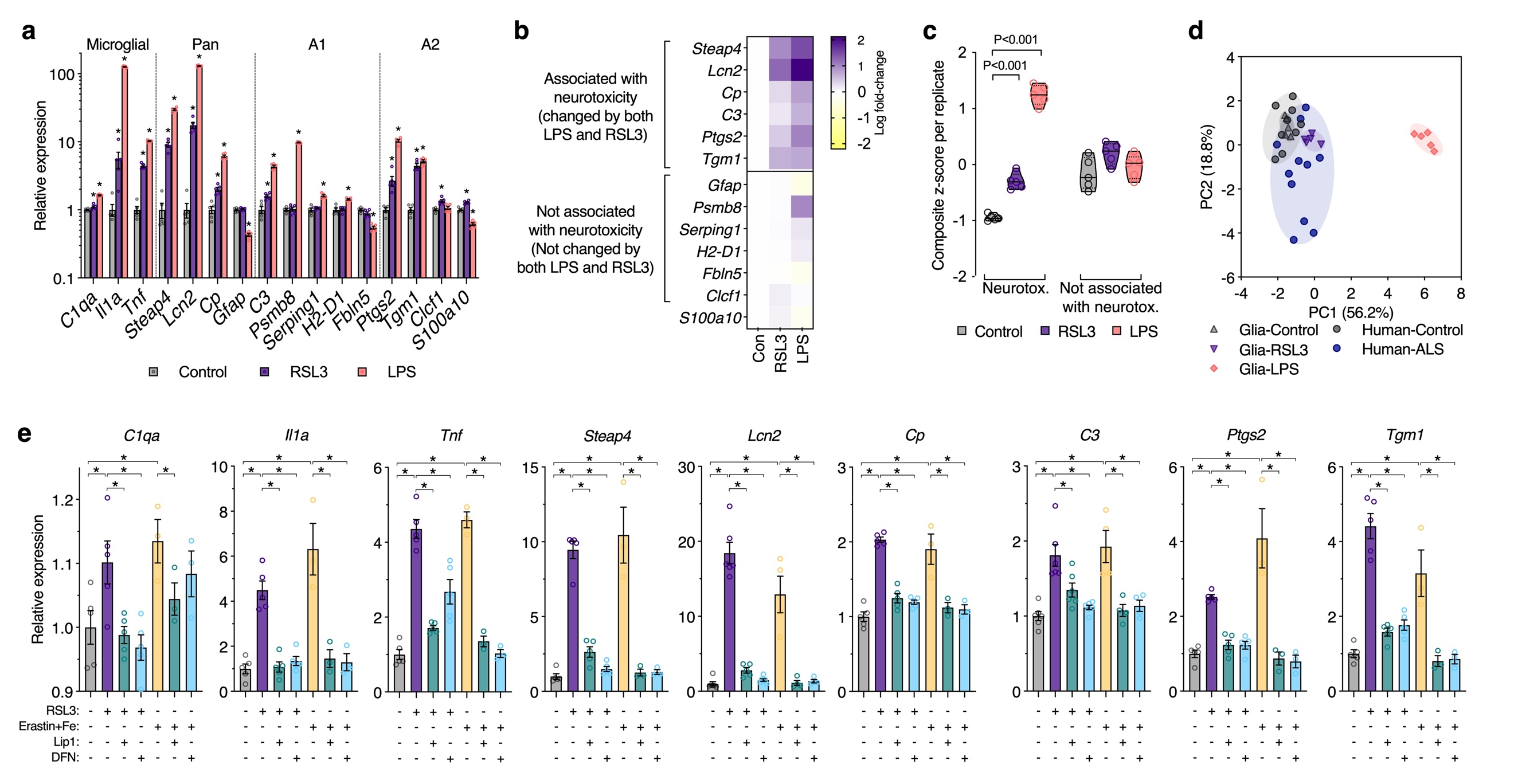
**

**Supplementary Figure 7. Gene expression changes in response to ferroptotic stress in glial cells.**

**(a)** Expression of genes associated with neurotoxic activation of glia in mixed glial cultures after treatment with RSL3 (200 nM) or LPS. Selected markers designated for microglial, pan, A1 and A2 activation are indicated. **(b-c)** Selection of genes used to monitor glial activation in response to ferroptotic stress. Activation genes that changed in response to both stressors and thus associated with neurotoxicity were chosen for subsequent analyses involving glial cultures exposed to LPS or inducers of ferroptotic stress. Violin plots in **c** represent overall transcript signature for features indicated, derived from heatmap data shown in **b**. **(d)** Principal component analysis of glial activation genes in response to RSL3 or LPS treatment in mixed glial cultures and human ALS-affected spinal cord. Symbols represent individual control and ALS cases analysed, or independent mixed glial cultures. Proportion of variance explained by each principal component is denoted on axes. Data are derived from fold expression change for all genes shown in **a** and **Supplementary Fig. 3c**. **(e)** Gene expression changes in mixed glial cultures treated with RSL3 (200 nM) or erastin plus iron (as ferric ammonium citrate) and effects of ferroptosis inhibitors liproxstatin-1 (Lip1) or deferiprone (DFN). Error margins are S.E.M. These data are presented in heatmap shown in **Fig. 3b**. Significant differences indicated by P values (**c**) or asterisks when P<0.05 (**a**,**e**) signifying differences between treatment groups and control (**a**) or as indicated (**e**).

**
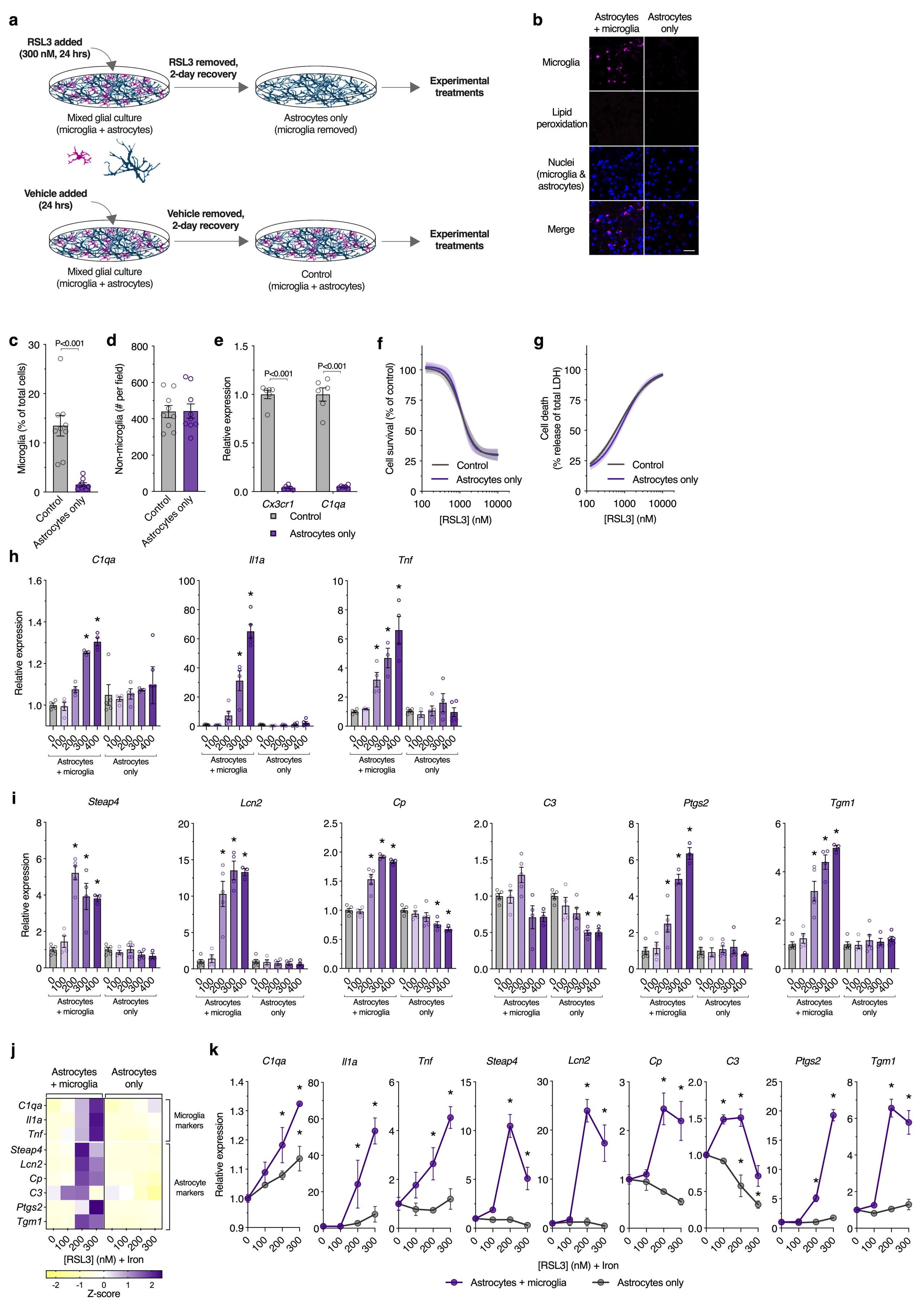
**

**Supplementary Figure 8. Isolating astrocytes from mixed glial cultures and gene expression changes in response to treatment with RSL3 or RSL3 plus iron.**

**(a)** Procedure to generate cultures of astrocytes without microglia from mixed glial cultures cultures (containing microglia and astrocytes) by treating with 300 nM RSL3 for 24 hrs. At this RSL3 dose, microglia are killed and astrocytes survive. The RSL3-containing medium is then removed, cultures washed, fresh medium applied, and astrocytes are allowed to recover for 2 days. Parallel treatment of mixed glial cultures with vehicle generates control cultures in which microglia are present. All analyses presented for isolated astrocytes are following RSL3 treatment and the 2-day recovery period. **(b)** Representative fluorescence microscopy images showing microglia and nuclei in control mixed glial cultures and cultures with microglia removed. Lipid peroxidation using C11-BODIPY is non-detectable at the end of the 2-day recovery period. Microglia (as detected by Dylight 649-labelled isolectin) are depicted as magenta, lipid peroxidation (oxidized:reduced C11-BODIPY) is yellow, and nuclei are blue. Scale bar = 50 μm. **(c-d)** Number of microglia (**c**) or non-microglial cells (**d**) as percentage of total cell population in mixed glial (control) or astrocyte cultures. **(e)** Expression of the microglial markers *Cx3cr1* and *C1qa* in mixed glial (control) or astrocyte cultures. **(f,g)** Cell survival (MTT reduction) and cell death (LDH release) in mixed glial (control) or astrocyte cultures in response to RSL3. These data show that the relatively low dose of RSL3 used to remove microglia does not alter subsequent sensitivity of the glial culture to RSL3 after the 2 day recovery period (n=4). **(h,i)** Gene expression changes in mixed glial and astrocyte cultures in response to indicated concentrations of RSL3. These data are presented in heatmap shown in **Fig. 3c**. **(j,k)** Expression of genes associated with microglial and astrocyte activation in response to indicated concentrations of RSL3 and transferrin-bound iron in mixed glial or astrocyte cultures (n=3-4). Individual heatmap values in **j** or symbols in **k** represent means. Data points represent independent cultures (**c-e**,**h**,**i**). Error margins are S.E.M. or 95% confidence intervals for fitted curves (**f**, **g**). P values indicate significant differences between treatment groups as indicated. Asterisks (denoting P<0.05) indicate significant differences between the indicated group and the respective no RSL3 group (**h**, **i**, **k**).


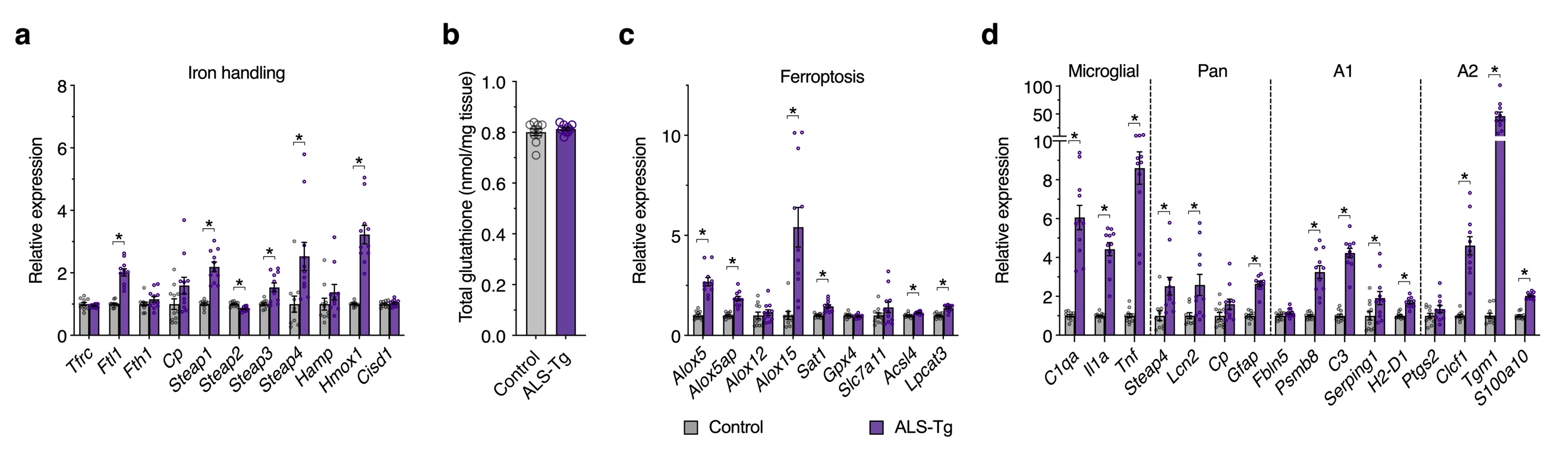


**Supplementary Figure 9. Glutathione and gene expression changes in spinal cord of SOD1^G37R^ mice compared to non-transgenic littermates.**

**(a-d)** Spinal cord tissue collected from SOD1^G37R^ mice (ALS-Tg) or non-transgenic littermates (Control) at 175-195 days of age, approximately 35-55 days post-symptom onset. **(a)** Relative expression changes for iron handling genes shown in heatmap in **Fig. 5c**. **(b)** Glutathione content. (**c)** Relative expression changes for ferroptosis-related genes shown in heatmap in **Fig. 5g**. **(d)** Relative expression changes for genes associated with neurotoxic glial activation shown in heatmap in **Fig. 5i** with markers for microglial, pan A1 and A2 activation indicated. Data points represent individual animals. Error bars represent S.E.M. Asterisks represent significant differences (P<0.05).


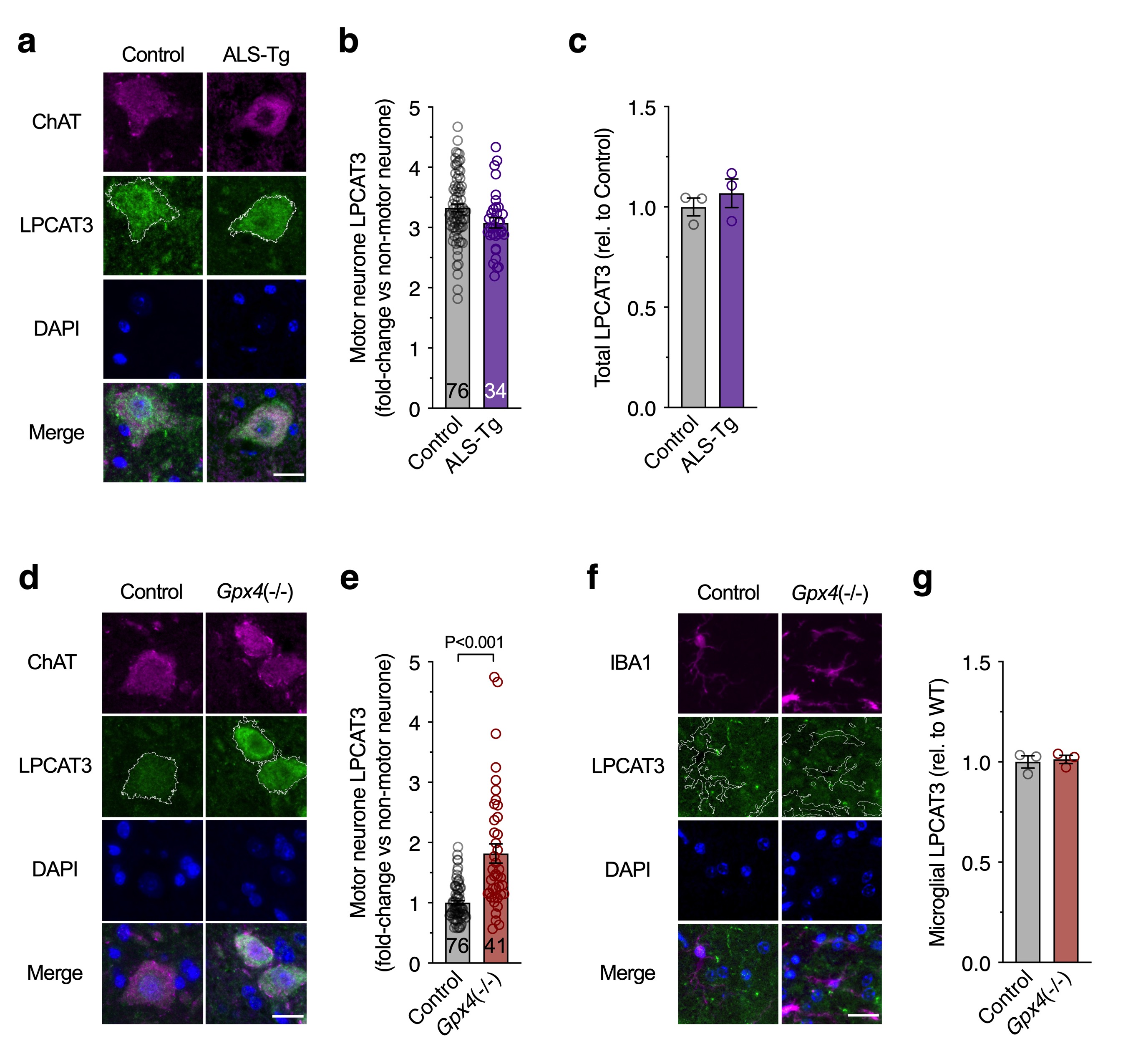


**Supplementary Figure 10. Changes in cellular expression of LPCAT3 in SOD1^G37R^ and *Gpx4*(-/-) mice.**

**(a-c)** Immunofluorescent analysis of spinal cord ventral horn tissue sections from SOD1^G37R^ mice (ALS-Tg) or non-transgenic littermates (Control) at 175-195 days of age, approximately 35-55 days post-symptom onset. **(a)** Representative immunofluorescence of ChAT-positive motor neurones (magenta) and LPCAT3 protein (green). **(b)** Motor neurone LPCAT3 quantitated from **a**. **(c)** Total LPCAT3 immunofluorescence across captured images. **(d-g)** Immunofluorescent analysis of spinal cord ventral horn tissue sections from control or *Gpx4*(-/-) mice. **(d)** Representative immunofluorescence of ChAT-positive motor neurones (magenta) and LPCAT3 protein (green). **(e)** Motor neurone LPCAT3 quantitated from **d**. **(f)** Representative immunofluorescence of IBA1-positive microglia (magenta) and LPCAT3 protein (green). **(g)** Microglial LPCAT3 quantitated from **f**. Data points are individual motor neurones (**b**,**e**) or individual animals (**c**,**g**). Outline of ChAT-positive motor neurones (**a**,**b**) or IBA1-positive microglia (**f**) are overlaid with LPCAT3. ChAT and LPCAT3 are false coloured in **a**,**d** for consistency with **f** and **Fig. 5**. Significant differences indicated by P values. Error bars represent S.E.M. Scale bar (**a**,**d**,**f**) = 20 µm.

**
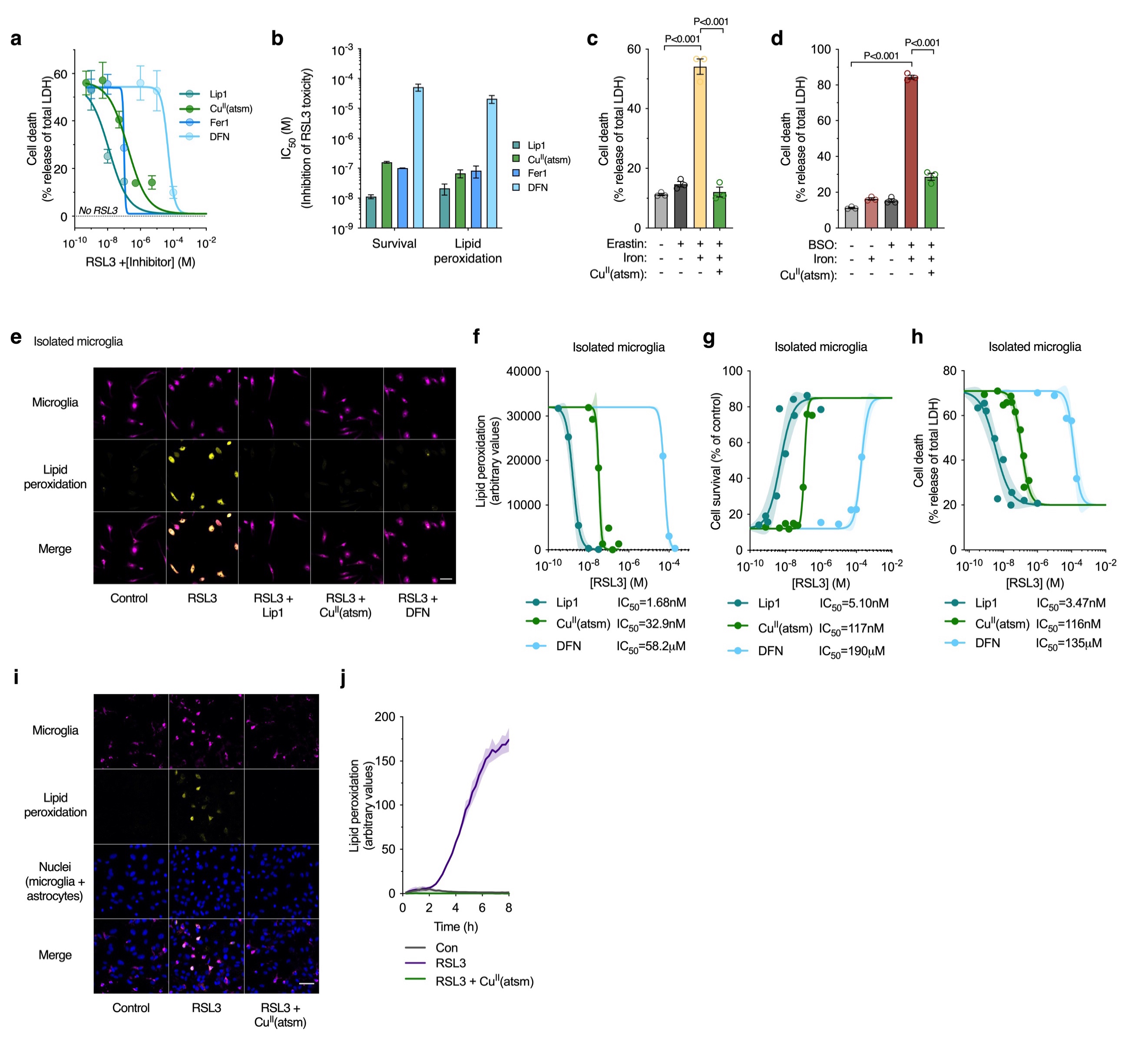
**

**Supplementary Figure 11. Protective activity of Cu^II^(atsm) *in vitro*.**

**(a)** Cu^II^(atsm) prevents RSL3-induced (2 µM) cell death (LDH release) in mixed glial cultures, with efficacy similar to the ferroptosis inhibitor ferrostatin-1 (Fer1) (n=3). **(b)** IC_50_ values (derived from data in **a** and **Fig. 6b**) for liproxstatin-1 (Lip1), Cu^II^(atsm), Fer1 and deferiprone (DFN) against RSL3 (n=2-4). **(c,d)** Cu^II^(atsm) prevents cell death (LDH release) in mixed glial cultures treated with ferroptosis inducers erastin plus iron and BSO plus iron (as ferric ammonium citrate). **(e)** Lipid peroxidation (visualised in **Supplementary Video 4**) in microglial cultures treated with RSL3 (2 µM) detected using C11-BODIPY (oxidized:reduced, yellow), is mitigated by Lip1, Cu^II^(atsm) and DFN. **(f-h)** Lipid peroxidation, cell survival (MTT reduction) and cell death (LDH release) in microglial cultures treated with RSL3 and Lip1, Cu^II^(atsm) or DFN. **(i,j)** Microglial lipid peroxidation in mixed glial cultures treated with RSL3 (100 nM) detected using C11-BODIPY (oxidized:reduced, yellow), is mitigated by Cu^II^(atsm). Time dependent changes in microglial lipid peroxidation (**j**) derived from **Supplementary Video 5** (n=1-3). Microglia in **e**,**i** detected with Dylight 649-labelled isolectin. Data points represent means (**a**, **f-h**) or independent cultures (**c**,**d**). Significant differences indicated by P values. Error margins are S.E.M. or 95% confidence intervals for fitted lines (**f-h**). Scale bars (**e**, **i**) = 50 μm.

**
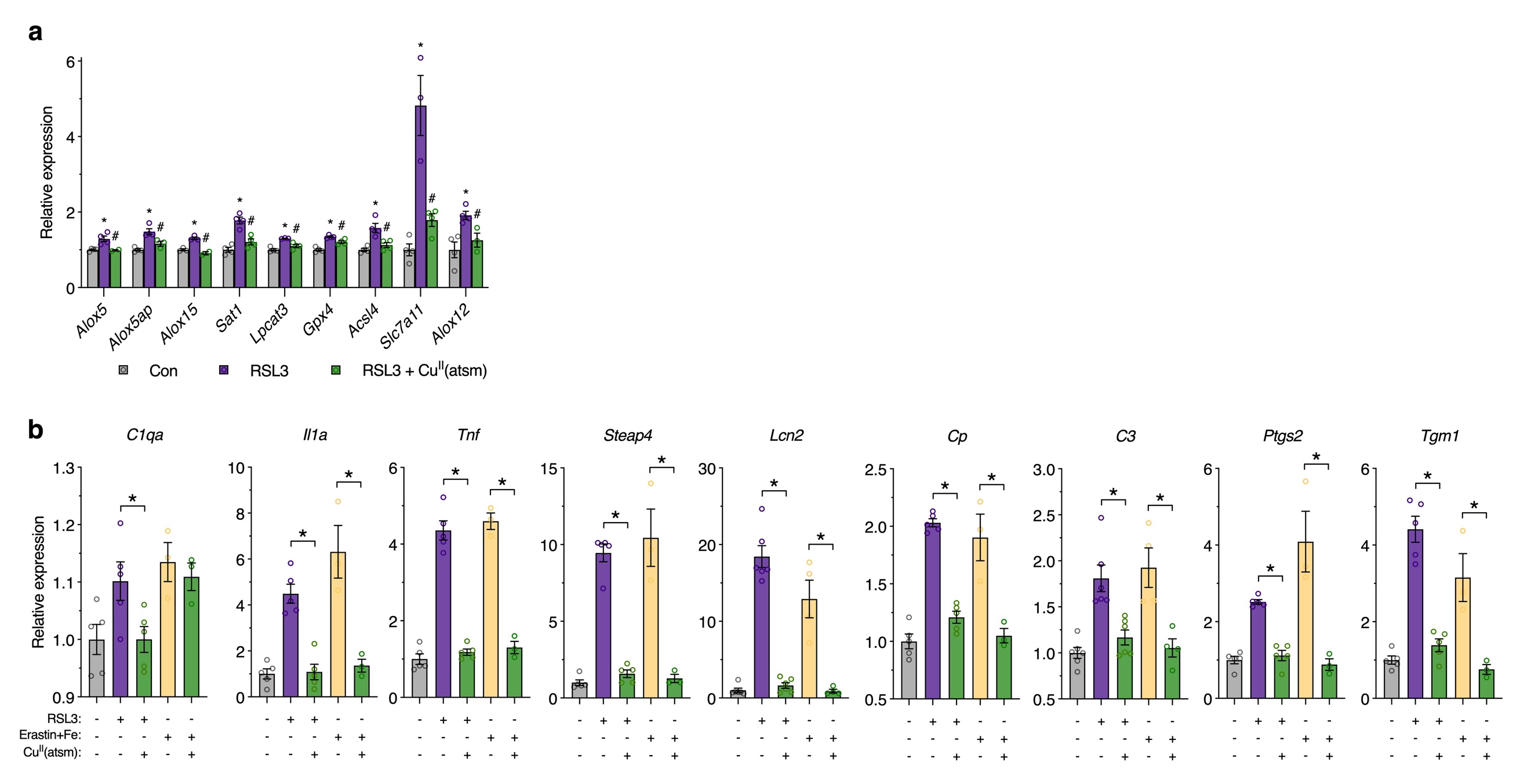
**

**Supplementary Figure 12. Gene expression changes in glial cultures treated with inducers of ferroptosis and Cu^II^(atsm).**

**(a)** Relative expression changes for individual genes in microglial cultures shown in heatmap in **Fig. 6c**. **(b)** Relative expression changes for individual genes in mixed glial cultures shown in heatmap in **Fig. 6d**. Asterisks illustrate significant differences (P<0.05) between RSL3 and control (**a**) or indicated treatment groups (**b**). Hash symbols represent significant differences (P<0.05) between RSL3 treated with or without Cu^II^(atsm). Data points represent independent cultures. Error margins are S.E.M.

**
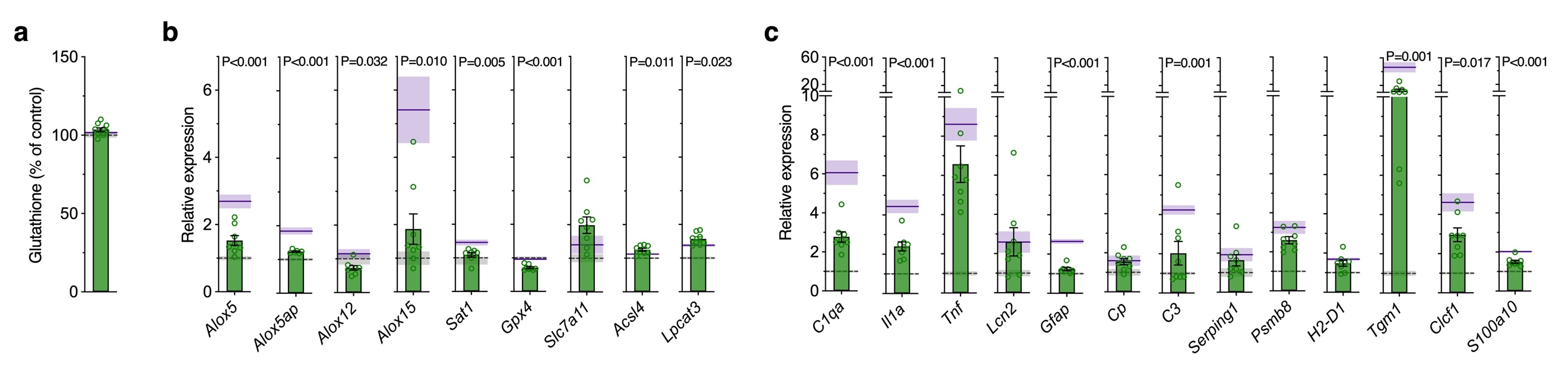
**

**Supplementary Figure 13. Effect of Cu^II^(atsm) on glutathione and gene expression changes in SOD1^G37R^ mice.**

**(a)** Glutathione content of spinal cord tissue from SOD1^G37R^ ALS model mice treated with Cu^II^(atsm), expressed as percentage of control non-transgenic littermates. **(b-c)** Expression changes for ferroptosis-related genes (**b**) and genes associated with neurotoxic glial activation (**c**) in spinal cord of SOD1^G37R^ ALS model mice treated with Cu^II^(atsm) expressed relative to non-transgenic littermates and shown in volcano plot in **Fig. 7i**. Data points represent individual SOD1^G37R^ mice treated with Cu^II^(atsm). Purple solid lines and grey dashed lines represent mean of SOD1^G37R^ mice without Cu^II^(atsm) treatment and non-transgenic littermates, respectively. P values indicate significant differences between SOD1^G37R^ mice treated with or without Cu^II^(atsm). Error margins represent S.E.M.

**
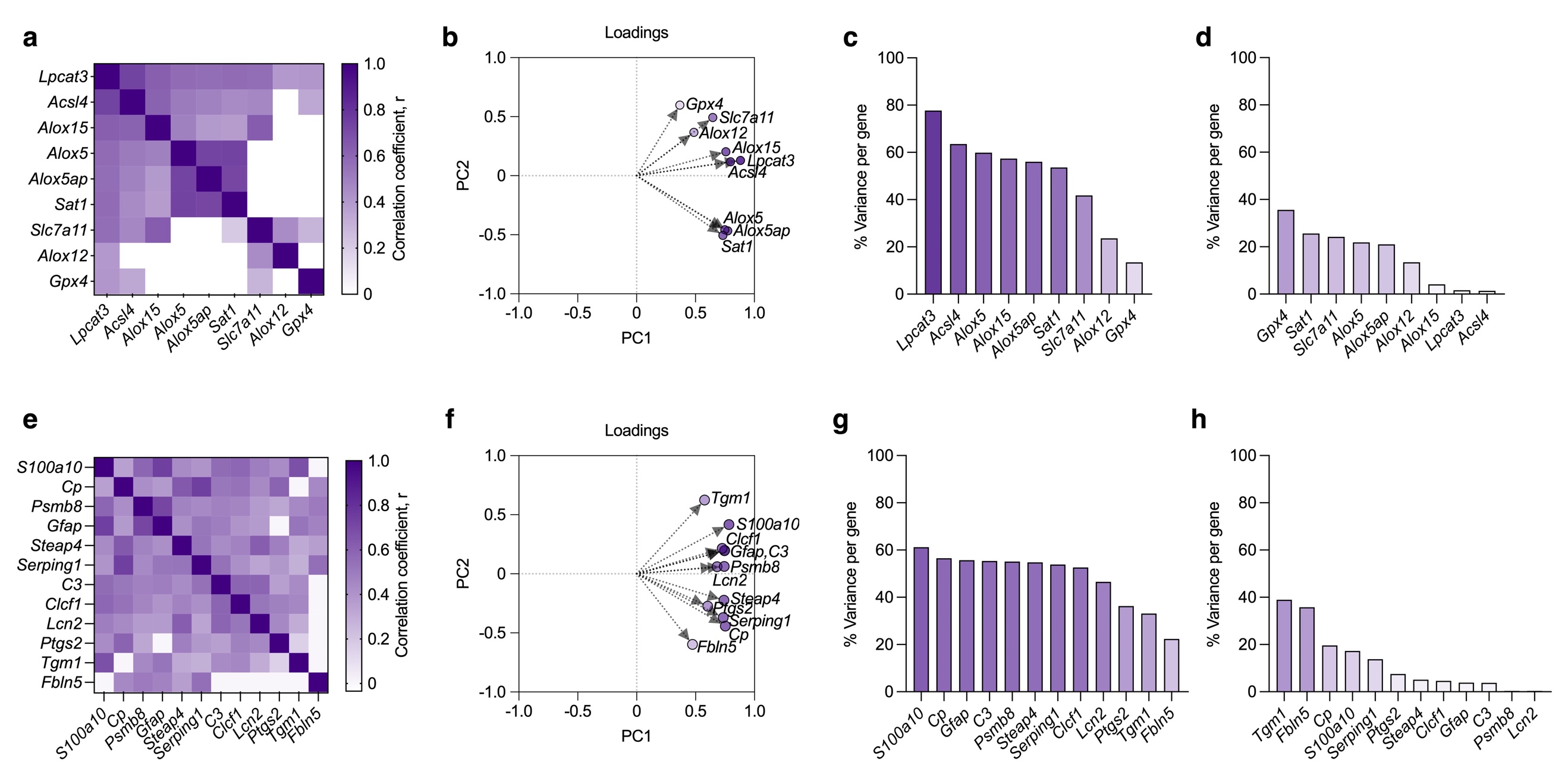
**

**Supplementary Figure 14. Analysis of PCA for gene expression changes in human ALS-affected spinal cord compared to SOD1^G37R^ mice and RSL3-treated glial cultures.**

**(a)** Correlation matrix for ferroptosis genes of combined human ALS cases, ALS model mice and RSL3-treated glial cultures and their controls. **(b)** Loading plot for PC1 and PC2 of ferroptosis genes. **(c-d)** Percentage of variance explained for each ferroptosis gene by their contribution to PC1 (**c**) or PC2 (**d**). **(e)** Correlation matrix for genes associated with neurotoxic glial activation of combined human cases and experimental models. **(f)** Loading plot for PC1 and PC2 of neurotoxic glial activation genes. **(g-h)** Percentage of variance explained for each neurotoxic glial activation gene by their contribution to PC1 (**g**) or PC2 (**h**). Percentage variance explained for each gene in a given PC (**c**,**d**,**g**,**h**) is calculated by the fraction of variance each gene contributes to that PC multiplied by the eigenvalue for that PC. This is equivalent to the r^2^ correlation of each gene to that PC. Data are derived from z-scores of expression changes shown in **Supplementary Fig. 3b**,**c**, **7a**, **9c**,**d** and **12a**. Correlations in **a**,**e** are excluded (set to zero) if not significant (P<0.05).
